# *Caenorhabditis* Intervention Testing Program: the tyrosine kinase inhibitor imatinib mesylate does not extend lifespan in nematodes

**DOI:** 10.17912/micropub.biology.000131

**Published:** 2019-07-03

**Authors:** Anna L. Coleman-Hulbert, Erik Johnson, Christine A. Sedore, Stephen A. Banse, Max Guo, Monica Driscoll, Gordon J. Lithgow, Patrick C. Phillips

**Affiliations:** 1 Institute of Ecology and Evolution, University of Oregon, Eugene, Oregon 97403, USA; 2 Division of Aging Biology, National Institute on Aging, Bethesda, Maryland 20892, USA; 3 Department of Molecular Biology and Biochemistry, Rutgers University, Piscataway, New Jersey 08854, USA; 4 The Buck Institute for Research on Aging, Novato, California 94945, USA

**Figure 1.**
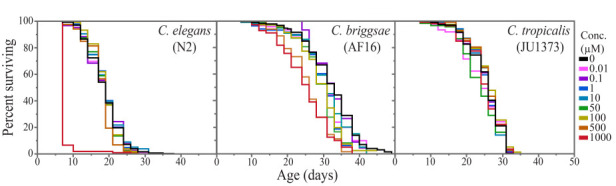
**Longevity under adult imatinib mesylate exposure.** Survival curves for *C. elegans* strain N2, *C. briggsae* strain AF16, and *C. tropicalis* strain JU1373 exposed to imatinib mesylate at various concentrations starting on day one of adulthood. Each line represents three technical replicates of 50 animals each. Mean lifespan of N2 on 1000 µM (mean=7.6 days, *p*<1e-04), AF16 on 50 µM (mean=29 days, *p*=0.0284), 500 µM (mean=25.6 days, *p*<1e-04), and 1000 µM (mean=24.9 days, *p*<1e-04), and JU1373 on 50 µM (mean=23.6 days, *p*=0.0361) differed significantly from the control (mean=18.5 days for N2, 32.1 days for AF16, 25.2 days for JU1373; Cox proportional hazard mixed-model using the coxme v.2.2-5 package in R (Therneau 2012)).

## Description

The *Caenorhabditis* Intervention Testing Program (CITP) is a National Institutes of Aging (NIA)-funded multi-institutional research consortium that investigates chemical interventions for their potential to extend lifespan and healthspan across a genetically diverse panel of *Caenorhabditis* nematodes (Lucanic *et al.,* 2017a). To date, the CITP has tested more than 20 compounds, including many of those previously surveyed in mice by the NIA-funded Interventions Testing Program (ITP) (Miller *et al.,* 2007; Nadon *et al.,* 2008). With compounds tested across multiple strains, species, labs, and concentrations, the CITP produces a large body of work before a compound traverses the full testing workflow. The intensive effort required poses an important challenge for the CITP as to how to systematically identify and prioritize compounds to test. Indeed, the selection of chemical interventions that might hold the greatest potential for efficacy is a challenge to the aging field in general.

One approach to chemical intervention prioritization is to use computational approaches that rank candidate compounds based on their likelihood to confer longevity benefits. For example, Ziehm *et al.* (2017) developed a ranking algorithm that combined information on genetic effects on aging, drug-target orthology relationships and sequence conservation, 3D protein structures, drug binding, and bioavailability. Here, we present the results of a test for lifespan effects of imatinib mesylate (a tyrosine kinase inhibitor; commercially known as Gleevec). Imatinib mesylate was the highest scoring drug-like compound with known mammalian targets ranked for likelihood to modulate aging in the invertebrates *C. elegans* and *D. melanogaster* (Ziehm *et al.,* 2017). Imatinib was also highly ranked by a separate computational approach that predicted compound interventions for human aging (Fuentealba *et al.,* 2019).

We assayed lifespan in response to imatinib mesylate exposure in three *Caenorhabditis* species in triplicate using our previously published workflow (Lucanic *et al.,* 2017a; b). In brief, imatinib mesylate (Toronto Research Chemicals) was dissolved in water and diluted appropriately such that 125 µl of solution could be added to 35 mm diameter NGM plates containing 51 µm FUdR in order to generate the following final imatinib mesylate concentrations: 0.01 µM, 0.1 µM, 1.0 µM, 10 µM, 50 µM, 100 µM, 500 µM, and 1 mM. Worms were age-synchronized by timed egg-lays on standard NGM plates, then transferred at a density of 50 animals per 35 mm plate (control or imatinib mesylate) in triplicate when they reached adulthood. Animals were maintained at 20 °C and moved to fresh experimental plates on the first, second, and fifth day of adulthood, then once weekly afterward, and fed on OP50-1 lawns for the duration of the experiments. Thrice weekly, we observed animals for spontaneous movement or movement after gentle perturbation with a 0.2 mm diameter platinum wire. Death was scored as a lack of movement.

Our results indicate that imatinib mesylate does not extend lifespan in any of the *Caenorhabditis* species at the concentrations tested here; in fact, at some concentrations, this compound reduced nematode lifespan, although this effect was not consistent among species (Fig. 1). However, as there was only a single biological replicate, this conclusion should be considered as preliminary. Interventions may be ineffective due to a range of causes including permeability barriers, compound stability *in vivo*, and metabolism by the bacterial food source. While we did not observe any lifespan-extending properties of imatinib mesylate in this study, it is important to note that the ranking methodology used to prioritize imatinib mesylate (Ziehm *et al.,* 2017) predicts whether compounds have aging-modulating effects rather than lifespan-extending properties *per se*. Notably, Ziehm *et al.* (2017) identified two aging-associated likely targets of imatinib, ABL1 and MAPK14. In *C. elegans,* *pmk-1* (MAPK14 ortholog) deletion mutants exhibit decreased survival compared to wild-type (Park *et al.,* 2018) indicating that inhibition by imatinib mesylate could likewise result in decreased lifespan. In contrast, *abl-1* (ABL1 ortholog) mutants are resistant to endoplasmic reticulum stress induced by tunicamycin (Judy *et al.,* 2013). Imatinib mesylate may target *abl-1* as it phenocopies *abl-1* mutants with respect to cell death phenotypes (Deng *et al.,* 2004).

In future studies, it will be important to further characterize whether compounds highly ranked in methodologies, such as the one discussed here, possess health or longevity-promoting effects as well as putative aging-modulating effects.

## Reagents

The following isolates were procured from the NIH Office of Research Infrastructure Programs-funded (P40 OD010440) *Caenorhabditis* Genetics Center (CGC): *C. elegans* N2_PD1073; *C. briggsae* AF16; *C. tropicalis* JU1373.
