# Corrigendum: Caenorhabditis Intervention Testing Program: the tyrosine kinase inhibitor imatinib mesylate does not extend lifespan in nematodes. microPublication Biology July 3, 2019. 10.17912/micropub.biology.000131

**DOI:** 10.17912/micropub.biology.000144

**Published:** 2019-07-31

## Description

The authors, Coleman-Hulbert, AL; Johnson, E; Sedore, CA; Banse, SA; Guo, M2; Driscoll, M3; Lithgow, GJ; and Phillips, PC, submit the following correction.

The first reference in the methods paragraph should be (Lucanic et al., 2017; Plummer et al., 2017) and should not be lettered accordingly.

“We assayed lifespan in response to imatinib mesylate exposure in three *Caenorhabditis* species in triplicate using our previously published workflow (Lucanic *et al.* 2017a; b).”

should be corrected to:

“We assayed lifespan in response to imatinib mesylate exposure in three *Caenorhabditis* species in triplicate using our previously published workflow (Lucanic et al., 2017; Plummer et al., 2017).”

