# Corrigendum: 10.17912/micropub.biology.000131

**DOI:** 10.17912/micropub.biology.000184

**Published:** 2019-10-25

## Description

The authors, Coleman-Hulbert, AL; Johnson, E; Sedore, CA; Banse, SA; Guo, M2; Driscoll, M3; Lithgow, GJ; and Phillips, PC, submit the following correction for 10.17912/micropub.biology.000131

The original text as read ““We assayed lifespan in response to imatinib mesylate exposure in three *Caenorhabditis* species in triplicate using our previously published workflow (Lucanic *et al.* 2017a; b).” is correct.

The reference Lucanic 2017b et al. is:

Lucanic M, Driscoll M, Plummer WT, Harke J, Chen E, Bhaumik D, Harinath G, Coleman-Hulbert A, Dumas K, Onken B, Johnson E, Fougler A, Guo S, Crist A, Presley M, Xue J, Sedore C, Chamoli M, Change M, Chen M, Angeli S, Royal MA, Willis J, Edgar D, Shobna P, Chao E, Kamat S, Hope J, Ibanez-Ventoso C, Kish J, Guo M, Phillips P, Lithgow G. Standardized protocols from the *Caenorhabditis* Intervention Testing Program 2013-2016: Conditions and assays used for quantifying the development, fertility and lifespan of hermaphroditic *Caenorhabditis* strains. Protoc. Exch. 2017. doi: 10.1038/protex.2016.086.​

The following reference is incorrect:

Plummer WT, Harke J, Lucanic M, Chen E, Foulger AC, Onken B, Coleman-Hulbert AL, Dumas KJ, Guo S, Johnson E, Bhaumik D, Xue J, Crist AB, Presley MP, Harinath G, Sedore CA, Chamoli M, Kamat S, Chen MK, Angeli S, Chang C, Willis JH, Edgar D, Royal MA, Chao EA, Shobna P, Garrett T, Ibanez-Ventoso C, Hope J, Kish JA, Guo M, Lithgow GJ, , Phillips PC. Standardized protocols from the Caenorhabditis Intervention Testing Program 2013-2016: Conditions and assays used for quantifying the development, fertility and lifespan of hermaphroditic Caenorhabditis strains. Protoc. Exch. 2017b. 10.1038/protex.2016.086

